# The Texas State Medical Association 1869-1901

**Published:** 1901-04

**Authors:** H. A. West

**Affiliations:** Secretary


### The Texas State Medical Association 1869-1901.

PRELIMINARY ANNOUNCEMENT AND PROGRAM OF THE THIRTY-THIRD ANNUAL MEETING, TO BE HELD IN GALVESTON, TEXAS, TUESDAY, WEDNESDAY, THURSDAY AND FRIDAY, APRIL 23D, 24TH, 25TH AND 26TH, 1901.

### COMMITTEES.

*Committee of Arrangements*—H. P. Cooke, Chairman.

*Committee of Entertainment*—Edward Randall, Chairman.

*Committee of Reception*—Geo. H. Lee, Chairman.

*Committee of Registration*—T. P. Lloyd, Geo. Delaney, J. T. Halley, W. P. Breath.

### OFFICERS.

*President*—B. E. Hadra....Dallas.
*First Vice-President*—Taylor Hudson....Belton.
*Second Vice-President*—Laurence Ashton....Dallas.
*Third Vice-President*—S. T. Turner....El Paso.
*Secretary*—H. A. West....Galveston.
*Treasurer*—R. F. Miller....Sherman.
*Orator*—Jno. O. McReynolds....Dallas.

### OFFICERS OF SECTIONS.

1. *General Medicine*—J. D. Law, Belton, Chairman; S. C. Red, Houston, Secretary.
2. *Obstetrics and Diseases of Children*—Wm. Keiller, Galveston, Chairman; O. L. Norsworthy, Houston, Secretary.
3. *Surgery*—I. N. Suttle, Corsicana, Chairman; J. S. Wooten, Austin, Secretary.
4. *Medical Jurisprudence*—J. S. Lankford, San Antonio, Chairman; J. H. Rawlins, El Paso, Secretary.
5. *State Medicine and Public Hygiene*—S. E. Milliken, Dallas, Chairman; J. B. Titterington, Dallas, Secretary.
6. *Gynecology*—Frank D. Thompson, Fort Worth, Chairman; J. M. Frazier, Belton, Secretary.
7. *Ophthalmology, etc.*—S. L. Terrell, Dallas, Chairman; W. L. Rogers, Galveston, Secretary.
8. *Pathology*—A. J. Smith, Galveston, Chairman; G. B. Foscue, Waco, Secretary.

### TRANSPORTATION.

All railroads in the State offer the following rates: Tickets will be sold to Galveston and return on the convention plan rate, providing for distances taking a higher rate than $3.00, one way, the round trip rate will be one fare plus ten per cent. For points taking a less rate than $3.00, the rate will be four cents per mile.

### HOTEL RATES.

Tremont, $2.00 to $4.00 per day; Grand $2.00 to $2.50 per day; Washington, $2.00 per day; New City, $1.50 per day.

### MEMORANDA.

1. Papers here announced will take precedence over those subsequently offered.

2. Papers will be read in the order as appears upon the program.

3. Papers read before the Association must be handed to the Secretary as soon as they shall have been read. Such papers, as well as those read by title, belong to the Association, but authors, if they so desire, may publish them elsewhere prior to publication in the Transactions.

4. Alterations will not be made in a paper after it is in type except at the expense of the author.

5. Illustrations will be inserted only at the expense of the author.

6. Papers read shall not occupy more than twenty minutes, except by permission. Discussion shall be limited to five minutes, unless the time be extended by unanimous consent of the sections, except as to the author of a paper, who shall have ten minutes in which to close.

No member shall speak more than once to the same subject without permission. (See By-Laws, Art. XV).

### PRELIMINARY PROGRAM.

The session will begin Tuesday morning at eleven o'clock. The place of meeting, Harmony Club, Postoffice, between 21st and 22d streets. Members and applicants are requested to assemble early in order to register, as far as possible, before the opening exercises. The Secretary, Treasurer and members of the Reception Committee will be present to aid registration. Applicants for membership, who belong to district or county societies in affiliation with the

State Association may join upon presentation of a certificate duly signed and accompanied with $5.00, one year's dues, and $2.50 initiation fee. Other applicants will sign blank form, giving post-office address and county. Each applicant must have the signature of two members and be accompanied by $7.50, covering one year's dues and the initiation fee. Such applicant must furnish diploma or other documentary evidence of graduation, for inspection by the Judicial Council. Badges will be furnished members and applicants after registration.

In accordance with a resolution adopted at San Antonio, requiring that the time for the meeting of the several sections to be printed in the program, and that all papers in a certain section be presented in their regular order at the time specified, the following is submitted:

PROVISIONAL ROSTER.

FIRST DAY—*Tuesday, April 23, 11 A. M.*

Opening exercises as follows:

Call to order by Chairman Committee of Arrangements, Dr. H. P. Cooke, Galveston.

Invocation, Rev. W. M. Harris, First Baptist Church.

Welcome, by Mayor W. C. Jones.

Welcome, by Judge Geo. E. Mann, representing Chamber of Commerce.

Welcome from Medical Profession of Galveston, Dr. Geo. H. Lee.

Response, Dr. B. E. Hadra, President.

Roll call.

Reading Minutes of Last Meeting.

Secretary's Annual Report, Dr. H. A. West.

Treasurer's Annual Report, Dr. R. F. Miller.

President's Annual Message and Recommendations, Dr. B. E. Hadra, Dallas.

*Afternoon Session, 2 o'clock.*

Medical Section.

*Night Session, 8 o'clock.*

Section on Obstetrics and Diseases of Children.

SECOND DAY—*Wednesday, 9 A. M.*

Executive Business.

Surgical Session.

*Afternoon Session, 2 P. M.*

Section on State Medicine.
Memorial Services.
Section Medical Jurisprudence.

*Night Session, 8 o'clock.*

Illustrated Lecture, "The Blood in Syphilis," L. H. Warner, M. D., New York City.
Section on Gynecology.

THIRD DAY—*Thursday, 9 A. M.*

*Morning Session.*

Executive Business.
Section on Ophthalmology and Pathology.

*Afternoon Session, 2 o'clock.*

Appointment of Nominating Committee.
Election of Officers.
Medical Section Recalled.

*Night Session, 8 o'clock sharp.*

President's Address, Dr. B. E. Hadra, Dallas.
Annual Oration, Dr. Jno. O. McReynolds, Dallas.

*9:30 o'clock.*

Reception, Harmony Club Rooms.

FRIDAY—*9 A. M., Morning Session.*

Papers not Previously Read in the Various Sections.
Executive Business.

GENERAL MEDICINE.

*Part I.*

1. Chairman's Address, J. W. Law, Belton.
2. "From Whence Comes the Microbe," J. M. Fort, Paris.
3. "Is Fever a Conservative or Non-Conservative Process," J. W. Embree, Belton.
4. "Acute Interstitial Nephritis, Pathology and Treatment," L. Ashton, Dallas.
5. "Further Observations on the Medical Aspects of the Galveston Storm," H. A. West, Galveston.

6. "Serum Therapy, Its Uses and Limitations," J. P. McElvey, Temple.

7. "Twentieth Century Smallpox," J. T. Benbrook, Rockwall.

8. "The Epidemic of Cerebro-Spinal Meningitis in Texas of 1898-99—Chiefly Statistical," I. J. Jones, Austin.

9. Title not Reported, W. R. Blailock, McGregor.

*Part II.*

Special Assignment.

10. "Malarial Infection, Its Prevention," Walter Shropshire, Yoakum.

11. "Malarial Hematuria," J. H. Sears, Waco.

Discussion opened by P. M. Raysor, Bryan.

SECTION ON OBSTETRICS AND DISEASES OF CHILDREN.

*Part I.*

1. Chairman's Address, "The Axis Traction Forceps," Wm. Keiller. Galveston.

2. "Puerperal Infection—Inquiry as to Sources and the Physician's Responsibility," Malone Duggan, Eagle Pass.

3. "Acute Septic Metritis," A. E. Spohn, Corpus Christi.

4. "Concealed Antepartum Hemorrhage," T. W. Shearer, Wallisville.

5. "Report on the Obstetrical Service of the John Sealy Hospital for the Ten Years Ending With 1900," J. F. Y. Paine, Galveston.

6. "Management of the Third Stage of Labor," B. F. Kingsley, San Antonio.

7. "Management of Woman During Labor," R. L. Howell, Chappell Hill.

*Part II.*

Special Assignment.

6. "Special Reference to the Technique of Suturing the Perineum," O. L. Norsworthy, Houston.

SURGERY.

1. Chairman's Address, I. N. Suttle, Corsicana.

2. "Treatment of Hare-Lip," J. E. Thompson, Galveston.

3. "Skin Grafting," A. E. Spohn, Corpus Christi.

4. "Chemical Treatment of Traumatisms," R. Harvey Reed, Rock Springs, Wyo.

5. "Report of a Case of Phelps' Open Operation for Tallipes Calcaneo Varus," W. Shropshire, Yoakum.

6. "Normal Salt Solution," A. C. Scott, Temple.

7. "Surgery of the Cranium and Brain. Report of Three Cases," H. A. Barr, Beaumont.

8. "Dry or Wet Dressings in Minor Surgery," S. T. Turner, El Paso.

9. "The Surgical Anatomy of the Ischio-Rectal Region and its Bearing on Piles and Fistula in Ano, With a New Operation for the Former," Wm. Keiller, Galveston.

10. Ectopic Testis. Report of a Case," G. W. Akard, Springtown.

STATE MEDICINE AND PUBLIC HYGIENE.

*Part I.*

Special Assignment.

1. Report of Chairman, S. E. Milliken, Dallas.

2. "Public Health and the State's Duty to Protect It," J. B. Massie, Houston.

*Part II.*

3. "Management of Epidemic Diseases," J. H. Florence, Dallas.

4. "Variations in Smallpox, as Seen in North Texas," Samuel W. Leeman, Honey Grove.

5. "Differential Diagnosis Between Smallpox and Syphilis," James B. Wilson, Dallas.

6. "Does Vaccination Prevent Smallpox," P. L. Campbell, Dallas.

7. "Contagious Diseases in Public Institutions," John S. Turner, Terrell.

MEDICAL JURISPRUDENCE.

1. Address of the Chairman, J. S. Lankford, San Antonio.

2. "Jury Trial of the Insane," Marvin L. Graves, San Antonio.

3. "An Ideal Eleemosynary System," John S. Turner, Terrell.

4. "Medical Jurisprudence as a Branch of State Medicine." R. H. Harrison, Columbus.

5. Importance and Difficulties of Insanity in its Medico-Legal Aspect," J. T. Wilson, Sherman.

GYNECOLOGY.

1. Report of Chairman, F. D. Thompson, Fort Worth.

2. "Endometritis with Especial Reference to the Use and Abuse of the Curette," J. E. Gilcreest, Gainesville. Discussion by Bacon Saunders, V. P. Armstrong and J. F. Y. Paine.

3. "Laparotomy for Fibroma of Ovary," G. W. Akard, Springtown.

4. "The Value of Calcium Carbide in the Treatment of Carcinoma of the Uterus," J. C. Chase, Fort Worth. Discussion opened by J. E. Gilcreest, Gainesville.

5. "Laceration of Cervix Uteri," J. E. Dodson, Vernon. To open discussion, J. F. Fields, Fort Worth.

6. "Ethyl Chloride Anæsthesia in Gynecology," T. J. Kennedy, Galveston.

7. "Obesrvations upon Trichomonas Vaginalis," T. J. Kennedy, Galveston.

*Part II.*

Special Assignment.

8. "Fibroid Tumors of the Uterus," A. E. Spohn, Corpus Christi.

9. "Fibroid Tumors of the Uterus—Report of Cases and Operation," J. H. Evans, Palestine.

OPHTHALMOLOGY, ETC.

1. "Lateral Pharyngeal Catarrh," Clarence Warfield, San Antonio.

2. "Relation of Disease of the Iris to Glaucoma," R. H. Chilton, Dallas.

3. "Lingual Tonsil—Report of a Case," F. D. Boyd, Fort Worth.

4. "Sympathetic Ophthalmia," Jno. O. McReynolds, Dallas.

5. "Mastoiditis—Report of a Case with Extra Dural Abscess," W. B. Anderson, Brownwood.

6. "Intubation vs. Tracheotomy," E. D. Capps, Fort Worth.

7. "Report of an Intubation Case with Complications," Joseph Mullen, Houston.

8. "Malarial Sequelæ of the Eye and Ear," H. W. Crouse, Victoria.

9. "The General Practitioner and the Specialist," W. R. Blailock, McGregor.

*Part II.*

Special Assignment.

10. "Simulated Blindness, its Detection,," H. L. Hilgartner, Austin. To open discussion, J. R. Ferrell, Waco.

PATHOLOGY.

The Chairman will distribute a number of blanks, requesting members to fill them in as fully as possible, bearing on the mosquito in relation to malarial fever. If sufficient information is obtained, he will tabulate results and report when the section is called.

1. "The Relation of the Parathyroids to the Thyroids," W. S. Carter, Galveston.
2. "Ankylostomiasis in Texas," M. C. Schaeffer, Galveston.
3. "Nephritis in Malarial Fever," J. T. Moore, Galveston.
4. "A Previously Undescribed Spore Forming Bacillus Associated with the Amœba in Hepatic Abscess," Allen J. Smith, Galveston.
5. "Spontaneous Amputation of Vermiform Appendix and Discharge Through the Bowels," J. M. Frazier, Belton.

The indications are favorable for a large attendance. The co-operation and attendance of all regular and reputable physicians in the State is earnestly solicited. The program is suggestive of a pleasant as well as profitable time.

Fraternally,

H. A. West, Secretary.

